# The Trimeric Artesunate Analog TF27, a Broadly Acting Anti-Infective Model Drug, Exerts Pronounced Anti-SARS-CoV-2 Activity Spanning Variants and Host Cell Types

**DOI:** 10.3390/pharmaceutics15010115

**Published:** 2022-12-29

**Authors:** Friedrich Hahn, Christina Wangen, Sigrun Häge, Lars Herrmann, Alexandra Herrmann, Svetlana B. Tsogoeva, Manfred Marschall

**Affiliations:** 1 Institute for Clinical and Molecular Virology, Friedrich-Alexander University of Erlangen-Nürnberg (FAU), Schlossgarten 4, 91054 Erlangen, Germany; 2 Organic Chemistry Chair I and Interdisciplinary Center for Molecular Materials (ICMM), Friedrich-Alexander University of Erlangen-Nürnberg, Nikolaus-Fiebiger-Straße 10, 91058 Erlangen, Germany; 3 Immunic AG, Lochhamer Schlag 21, 82166 Gräfelfing, Germany

**Keywords:** SARS-CoV-2 infection, host-directed drug targeting, artesunate and analogs, trimeric analog TF27, broad-spectrum inhibitory properties, pronounced anti-SARS-CoV-2 activity

## Abstract

Starting in 2019, the spread of respiratory syndrome coronavirus 2 (SARS-CoV-2) and the associated pandemic of the corona virus disease (COVID-19) has led to enormous efforts in the development of medical countermeasures. Although innovative vaccines have scaled back the number of severe COVID cases, the emergence of the omicron variant (B.1.1.529) illustrates how vaccine development struggles to keep pace with viral evolution. On the other hand, while the recently approved antiviral drugs remdesivir, molnupiravir, and Paxlovid are considered as broadly acting anti-coronavirus therapeutics, only molnupiravir and Paxlovid are orally available and none of these drugs are recommended for prophylactic use. Thus, so far unexploited small molecules, targeting strategies, and antiviral mechanisms are urgently needed to address issues in the current pandemic and in putative future outbreaks of newly emerging variants of concern. Recently, we and others have described the anti-infective potential and particularly the pronounced antiviral activity of artesunate and related compounds of the trioxane/sesquiterpene class. In particular, the trimeric derivative TF27 demonstrated strong anti-cytomegalovirus activity at nanomolar concentrations in vitro as well as in vivo efficacy after oral administration in therapeutic and even prophylactic treatment settings. Here, we extended this analysis by evaluating TF27 for its anti-SARS-CoV-2 potential. Our main findings are as follows: (i) compound TF27 exerted strong anti-SARS-CoV-2 activity in vitro (EC_50_ = 0.46 ± 0.20 µM), (ii) antiviral activity was clearly distinct from the induction of cytotoxicity, (iii) pretreatment with TF27 prevented virus replication in cultured cells, (iv) antiviral activity has likewise been demonstrated in Calu-3 human lung and Caco-2 human colon cells infected with wild-type, delta, or omicron SARS-CoV-2, respectively, and (v) analysis of TF27 combination treatments has revealed synergistic interaction with GC376, but antagonistic interaction with EIDD-1931. Combined, the data demonstrated the pronounced anti-SARS-CoV-2 activity of TF27 and thus highlight the potential of trioxane compounds for further pharmacologic development towards improved options for COVID-specific medication.

## 1. Introduction

The rapid spread of severe respiratory syndrome coronavirus 2 (SARS-CoV-2) within the human population worldwide causing the still ongoing pandemic has led to unprecedented speed in the development and approval of direct-acting antivirals (DAAs). These include remdesivir and molnupiravir, two nucleoside analogs targeting viral genome replication, and Paxlovid which contains the active determinant nirmatrelvir, an inhibitor of the viral protease 3CLpro, supplemented by the half-life boosting ritonavir. Despite their markedly potent antiviral activity in vitro and success in the clinical context, these DAAs however face several limitations in clinical settings. These include the exclusive intravenous application of remdesivir and the mutagenesis-based mechanism of molnupiravir that might promote viral evolution with the risk of generating viral variants that are not only drug-resistant, but also evade host adaptive immunity [1]. Moreover, molnupiravir has been shown to introduce mutations into the human genome in vitro [2]. Ritonavir contained in the formulation of Paxlovid also affects the pharmacokinetics of numerous other drugs, which complicates treatment decisions in patients with co-medications to avoid adverse drug events. Therefore, it is crucial to identify novel antiviral drug candidates that are orally available, avoid resistance formation, and are accessible to combination therapy.

Artemisinin, its semisynthetic drug artesunate (ART), and related chemical derivatives were originally developed and are clinically applied as a potent and well-tolerated antimalarial medication. In the last decade, it has been repeatedly reported that this class of compounds possesses additional activities against human diseases including cancer, diabetes, and viral infections [3,4,5,6,7]. The antiviral activity of ART and related compounds was particularly demonstrated for human cytomegalovirus (HCMV) [8,9,10], a number of additional herpesviruses [6,11,12], and even other pathogenic human viruses [7,13,14,15,16,17]. Based on the initial promising anti-HCMV activity of ART, a 10- to 100-fold improvement in antiviral activity was achieved by applying a chemical hybridization concept to yield ART multimers, i.e., compounds that combine coupled trioxane moieties [10,18,19,20], or conjugates of ART to other bioactive compounds [18,21,22,23,24,25]. In particular, the trimeric ART derivative TF27 stood out for its pronounced antiviral activity in a nanomolar range in vitro and displayed potent in vivo therapeutic as well as prophylactic efficacy [12,26,27].

In our present study, we demonstrate that TF27 inhibited SARS-CoV-2 replication more effectively when compared to the parental compound ART in conventional treatment schemes and exclusive pretreatment settings. Additionally, by using the Loewe additivity fixed-dose method, potentially synergistic drug interactions with direct-acting anti-SARS-CoV-2 drugs, i.e., a viral protease inhibitor and a nucleoside analog, were investigated. Thus, the findings underline the outstanding antiviral potential of the candidate drug TF27 and highlight the chances to further develop the chemical class trioxane/sesquiterpenes for future SARS-CoV-2-specific treatment options.

## 2. Materials and Methods

### 2.1. Antiviral Compounds

The protocol for TF27 synthesis has been detailed previously [10,20]. A new batch was synthesized by Vichem Chemie Research Ltd. (Budapest, Hungary). Artesunate (ART) was purchased from Saokim Ltd. (Hanoi, Vietnam). EIDD-1931 was kindly provided by Immunic AG, Gräfelfing, Germany. GC376 was purchased from TargetMol (Boston, MA, USA). Stock aliquots were prepared in pure DMSO (Sigma, St. Louis, MO, USA) at 10 mM and stored at −20 °C.

### 2.2. Cultured Cells, SARS-CoV-2 Isolates and Reporter Viruses, and Fluorescence-Based and Multi-Readout Replication Assays

For SARS-CoV-2 infection experiments, human Caco-2 colon cells and Calu-3 lung cells were cultivated at standard conditions and infected with either (i) the SARS-CoV-2 d6-YFP recombinant reporter virus, (ii) the wild-type isolate MUC-IMB-1/2020, or (iii–iv) the variants of concern (VOCs) delta and omicron. The generation and isolation of virus stocks, the amount of viral inocula applied and the incubation periods for the different replication settings are detailed in the Supplementary Methods and have in parts also been described in previous works [22,28,29]. Replication was terminated using 10% formalin as fixative. For d6-YFP, replication was assessed by quantitation of the intracellular fluorescence of the YFP reporter. For the non-labeled viruses, the cells were permeabilized and the infection rates were determined by antibody-mediated detection of replication-associated antigens using pAb-SARS-CoV-2-nsp3 (PA5116947, ThermoFisher Scientific, Waltham, MA, USA) or mAb-S TRES-6.18 [22,30] in combination with the secondary antibody anti-mouse Alexa 488 (A11029, ThermoFisher Scientific), as indicated. Fluorescence quantitations were performed in a Victor X4 microplate reader (PerkinElmer, Waltham, MA, USA). In specific cases, the antiviral activity was determined by antibody- and RT-qPCR-based measurements of several SARS-CoV-2 replication parameters within our previously established multi-readout assay (MRA) as described in the Supplementary Methods [22,31]. The inhibitory activity of compounds on SARS-CoV-2 replication was defined as the reduction in virus replication in compound-treated cells relative to solvent-treated control cells (DMSO), resulting in the arithmetic mean values ± SD of biological quadruplicates as described earlier [22,29]. All of the SARS-CoV-2 infection experiments were performed under BSL-3 conditions. As counter screens for compound-induced toxicity induction, Neutral Red uptake assays (NRA) and lactate dehydrogenase release assays (LDH) were performed as described previously [22,28,32] and are also detailed under Supplementary Methods.

### 2.3. Assessment of Drug Interactions Using the Loewe Additivity Fixed Dose Method

Drug interactions were determined by the Loewe additivity method using the d6-YFP-based replication assay as described previously [22] and are listed in the Supplementary Methods.

## 3. Results

### 3.1. The Trimeric Artesunate Analog TF27 Inhibits SARS-CoV-2 Replication in Caco-2 Cells

**Figure 1:**
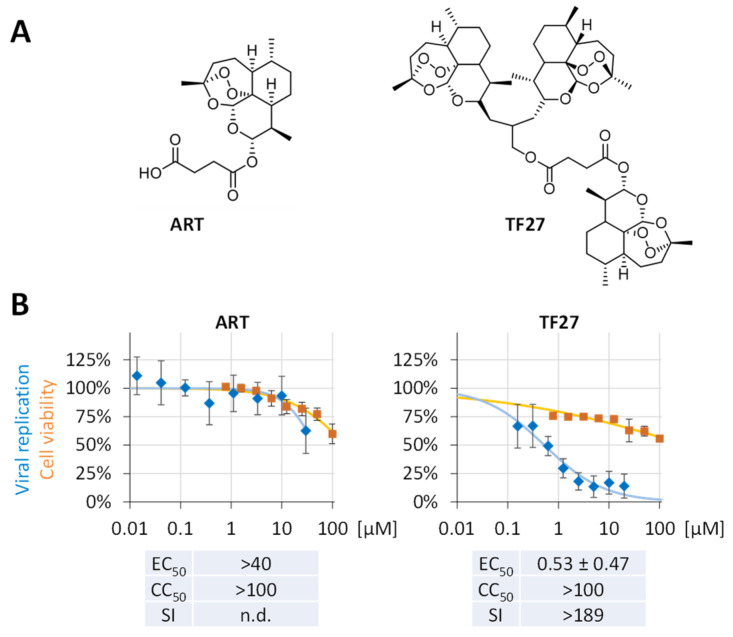
Anti-SARS-CoV-2 activity of ART and its optimized derivative TF27. (**A**) Chemical structures depicting artesunate (ART) and its trimeric derivative TF27. (**B**) Human Caco-2 cells cultivated in 96-well plates at 25,000 cells per well were infected with the recombinant SARS-CoV-2 reporter virus d6-YFP at the MOI of 0.003 and treated with the indicated concentrations of compounds. At 28 h p.i., the cells were fixed using formalin and viral replication was determined by quantitation of the cell-associated YFP fluorescence. Cell viability was measured in parallel cultures of uninfected Caco-2 cells after 48 h of treatment by Neutral Red assay (NRA). The values represent means ± SD of the biological quadruplicates (viral replication) or triplicates (NRA/cell viability).

Previously, we described a variety of novel artesunate (ART)/artemisinin-based compounds including hybrids and multimers regarding their activity against the replication of the diverse herpesviruses in vitro including human (HCMV), murine cytomegalovirus (MCMV) in vivo, and other non-herpes viruses (Table 1). In particular, TF27 comprises three artemisinin/trioxane moieties connected via a chemical linker to yield a trimeric structure and represents one of our most active ART compounds (Figure 1A) [10,18,19,20]. Several studies from other researchers have recently reported the initial in vitro anti-SARS-CoV-2 activity of ART and related monomeric compounds, albeit with limited to intermediate efficacies at micromolar concentrations [33,34,35,36,37,38]. These indications prompted us to investigate our optimized ART derivative TF27 and its anti-SARS-CoV-2 in vitro efficacy compared to the inhibitory effects described previously for HCMV/herpesviral replication [10,20]. To this end, TF27 and the parental compound ART were analyzed in parallel. As an established replication model, we used human Caco-2 cells infected with the recombinant SARS-CoV-2 reporter virus d6-YFP, which expresses the yellow fluorescent protein (YFP) replacing the viral ORF6 [22,29]. The effective drug concentrations of half-maximal inhibition (EC_50_) of SARS-CoV-2 replication were derived from the dose-specific response for ART and TF27 (Figure 1B, Table 2). While the parental compound ART only achieved partial inhibition of virus replication and did not reach the EC_50_ up to 40 µM, TF27 revealed a pronounced anti-SARS-CoV-2 effect with a submicromolar EC_50_ value of 0.53 ± 0.47 µM that was at least a 75-fold increase over ART (Figure 1B, Table 2).

**Figure 2:**
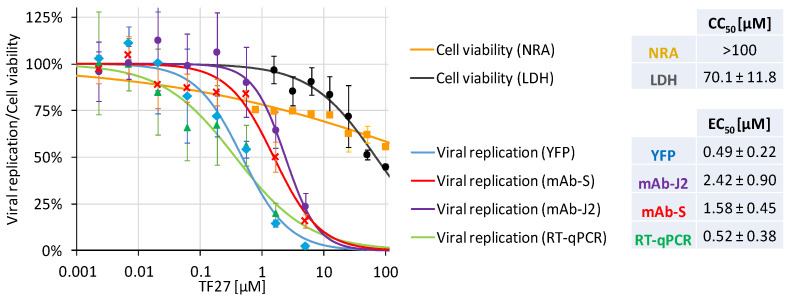
Anti-SARS-CoV-2 activity of TF27 analyzed by multiple parallel readouts. Human Caco-2 cells were infected with SARS-CoV-2 d6-YFP at the MOI of 0.003 and treated with the indicated concentrations of TF27. At 28 h p.i., cells were fixed using formalin and viral replication was determined by quantitation of the cell-associated YFP fluorescence (YFP), antibody stainings for viral dsRNA (mAb-J2) or the viral spike protein (mAb-S), and detection of released viral genomes using the cell culture supernatant (RT-qPCR). Cell viability was measured in parallel cultures of uninfected Caco-2 cells after 48 h of treatment by Neutral Red assay (NRA) as well as lactate dehydrogenase release assay (LDH). The values represent means ± SD of the biological quadruplicates (replication) or triplicates (NRA and LDH).

To characterize the antiviral effect of TF27 in greater detail, viral replication was analyzed by different methods besides the d6-YFP reporter. This included analyses of dsRNA as an intermediate of viral genome replication, viral spike (S) protein synthesis, and virus release into the cell culture media by genome-specific RT-qPCR (Figure 2). This approach resulted in comparable or slightly increased EC_50_ values compared to the YFP reporter (Figure 2, panels YFP, mAb-J2 [44], mAb-S [30] and RT-qPCR). These differences are a likely consequence of assessing separate markers of viral replication which exhibit their distinct kinetics within the viral replication cycle. To discriminate between antiviral effects and drug-mediated cell damage, cell viability was additionally monitored by lactate dehydrogenase (LDH) release assay in addition to the NRA. Whereas the uptake of Neutral Red in the NRA is also reduced under conditions that are not necessarily equivalent to cytotoxicity induction, e.g., in the case of compounds with antiproliferative effects, the LDH release assay specifically detects cell damage by addressing the integrity of the plasma membrane. Consequently, both assays provide complementary information on cell viability, indicating TF27-CC_50_ values of 70.1 ± 11.8 µM (LDH) and >100 µM (NRA). These data led to SI values (EC_50_/CC_50_) of 29 to >204 and thus demonstrate remarkable separation of antiviral activity from cytotoxicity induction. Combined, these results underline that our trioxane multimerization approach used to generate TF27 and additional ART dendrimers does not only yield optimized antiviral drug candidates for HCMV as previously demonstrated, but also for SARS-CoV-2 as exemplified here for TF27.

### 3.2. Pretreatment Efficacy Demonstrates the Targeting of Host Cell Proteins

**Figure 3:**
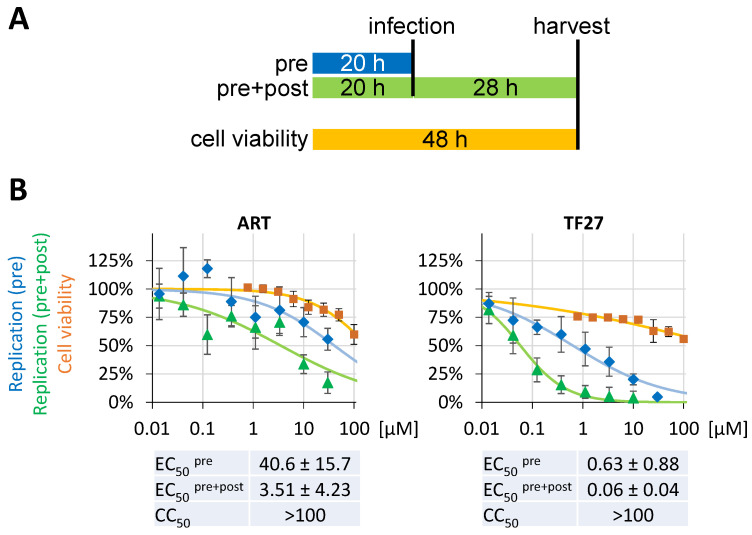
Anti-SARS-CoV-2 activity exerted by pretreatment of cells with TF27. (**A**) Schematic depiction of the structured treatments. (**B**) Human Caco-2 cells cultivated in 96-well plates at 25,000 cells per well were pretreated with indicated concentrations of ART or TF27. After 20 h, compound-containing supernatants were discarded and the cells were either infected under post-infection treatment in the continued presence of compounds (pre+post) in the same concentrations used for pretreatment, or the cells were subjected to a wash-out before virus infection and further cultivated in the absence of antiviral compounds (pre). In both cases, the recombinant SARS-CoV-2 reporter virus d6-YFP was used at the MOI of 0.003. At 28 h p.i., the cells were fixed using formalin and viral replication was determined by quantitation of the cell-associated YFP fluorescence. The values represent means ± SD of the biological quadruplicates (replication) or triplicates (NRA).

Numerous studies have illustrated that ART-derived compounds exert their main biological activity through the covalent alkylation of multiple target proteins by the reaction of the ART endoperoxide bridge with cysteine residues [42,45,46,47,48,49]. This prompted us to ask whether the inhibitory activity of ART-derived compounds either relies on the steady presence of the drug in solution or on covalently modified target proteins. Supporting this idea, we have previously demonstrated that TF27 maintains nearly 50% of its anti-HCMV activity even when cells are pretreated followed by a wash-out [27]. Moreover, an analogous prophylactic effect was confirmed for MCMV-infected mice [27]. Therefore, Caco-2 cells were pretreated for 20 h, followed by a wash-out, prior to infection with SARS-CoV-2 in the absence of compounds (Figure 3A). Consistent with our previous findings for HCMV, TF27 inhibited viral replication with an EC_50_ of 0.63 ± 0.88 µM even under conditions of exclusive pre-treatment (Figure 3B, pre). The effectiveness of the wash-out procedure was confirmed in parallel experiments with a 1 h incubation period, since this treatment plus wash-out did not exhibit a measurable decrease in viral replication, in contrast to the 20 h incubation plus wash-out (Figure 3B, right panel). Moreover, when pre-treatment was combined with the conventional post-infection treatment setting, even the very poorly active ART significantly reduced viral replication with an EC_50_ of 3.51 ± 4.23 µM (Figure 3B, left panel). In this treatment scheme of pre+post, the EC_50_ of TF27 further improved in efficacy down to 0.06 ± 0.04 µM. Taken together, TF27 and to some extent ART exert anti-SARS-CoV-2 activity when exclusively administered pre-infection, an effect that is further enhanced upon post-treatment to achieve a mid-nanomolar EC_50_ of TF27.

### 3.3. TF27 Inhibits SARS-CoV-2 Replication in Calu-3 Human Lung Cells and Is Active against Clinical Isolates including Delta and Omicron Variants

**Figure 4:**
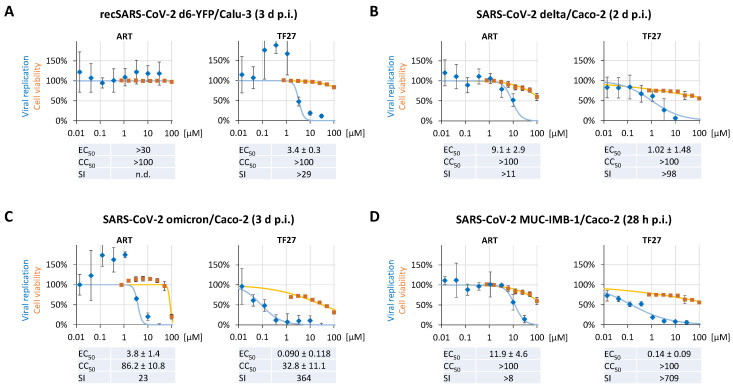
TF27 exerts anti-SARS-CoV-2 activity in two different human host cell types and is similarly effective against viral delta and omicron variants as well as the MUC-IMB-1/2020 isolate. (**A**) Human Calu-3 lung cells cultivated in 96-well plates at 25,000 cells per well were infected with the recombinant SARS-CoV-2 reporter virus d6-YFP at the MOI of 0.003 and treated with the indicated concentrations of compounds. At 3 d p.i., the cells were fixed using formalin and viral replication was determined by quantitation of the cell-associated YFP fluorescence. (**B**) Human Caco-2 cells cultivated in 96-well plates at 25,000 cells per well were infected with the SARS-CoV-2 delta variant; (**C**) in parallel, Caco-2 cells were infected with the SARS-CoV-2 omicron variant. In both cases, the inoculum used had been empirically determined to achieve 75% infected cells at the time point of harvest. (**D**) Caco-2 cells cultivated in 96-well plates at 25,000 cells per well were infected with the SARS-CoV-2 MUC-IMB-1/2020 isolate at an MOI of 0.003. The infected cells were treated with the indicated concentrations of compounds. At 2 d p.i. (delta), 3 d p.i. (omicron), or 28 h p.i. (MUC-IMB-1/2020), the cells were fixed using formalin and viral replication was determined by staining with the monoclonal antibody recognizing the SARS-CoV-2 nonstructural protein 3 (delta and omicron) or the viral spike protein (MUC-IMB-1/2020) followed by incubation with a fluorescently labeled secondary antibody. Viral replication was assessed by quantitation of the cell-associated fluorescence. Cell viability was measured for each infection setting using parallel cultures of uninfected cells from the respective cell type incubated with ART or TF27 for a duration matching at least the period of replication. Cell viability was determined by Neutral Red assay (NRA). The values represent means ± SD of the biological quadruplicates (replication) or triplicates (NRA).

A host factor-centered targeting mode, such as the mode observed for ART compounds (host-directed antivirals, HDAs), is generally considered to impose a high barrier towards viral resistance formation. It is also considered to be efficacious against viruses resistant to direct-acting antivirals (DAAs). Thus, such a HDA-targeting mechanism conversely raises the question as to whether the anti-SARS-CoV-2 activity is preserved among distinct permissive cell types. Therefore, infection experiments with the Calu-3 human lung cell line were performed to refer to the antiviral effects determined with the abovementioned Caco-2 cells. Treatment of d6-YFP-infected Calu-3 cells with TF27 exerted a marked block of SARS-CoV-2 replication, whereby the resulting EC_50_ of 3.4 ± 0.3 µM was approx. 7-fold higher than in Caco-2 cells (Figure 4A). Interestingly, concentrations between 0.1 and 1 µM reproducibly increased viral replication, a phenomenon that was not observed for ART. Nevertheless, significant levels of inhibition were achieved with increasing concentrations above 1 µM, confirming the potency of TF27 in this SARS-CoV-2-infected cell type. As a second aspect of the HDA-associated mechanism, we expected that TF27 would possess broad anti-SARS-CoV-2 activity, including against SARS-CoV-2 variants of concern (VOCs) and even other coronaviruses as well. To address this issue, we analyzed the effect of TF27 on the replication of the delta and omicron variants in Caco-2 cells. It is worth mentioning that these isolates differ in their replication behavior when compared to d6-YFP. Whereas replication of delta leads to a rapid formation of syncytial polynucleated cells, the omicron isolate barely induces cell–cell fusion. Additionally, the overall spread of omicron occurs over an extended period of time in Caco-2 cells, a finding consistent with other reports [50,51]. Despite these individual characteristics of viral replication, TF27 inhibited delta and omicron with EC_50_ values of 1.02 ± 1.48 µM and 0.090 ± 0.118 µM, respectively (Figure 4B,C). In our comparative analysis, including the Wuhan-like wild-type SARS-CoV-2 isolate MUC-IMB-1/2020, data indicated very consistent anti-SARS-CoV-2 activity between the wild-type and variants. Here, the EC_50_ was 0.14 ± 0.09 µM of TF27 (11.9 ± 4.6 µM of ART) for MUC-IMB-1/2020, as the mean value derived from the measurement of three biological replicates (Figure 4D). Taken together, comparative analysis in different cell types and by using different virus strains revealed broad anti-SARS-CoV-2 activity with low micromolar to submicromolar EC_50_ values.

### 3.4. TF27 Exhibits Synergistic Interaction with GC376 but Antagonistic Interaction with EIDD-1931

**Figure 5:**
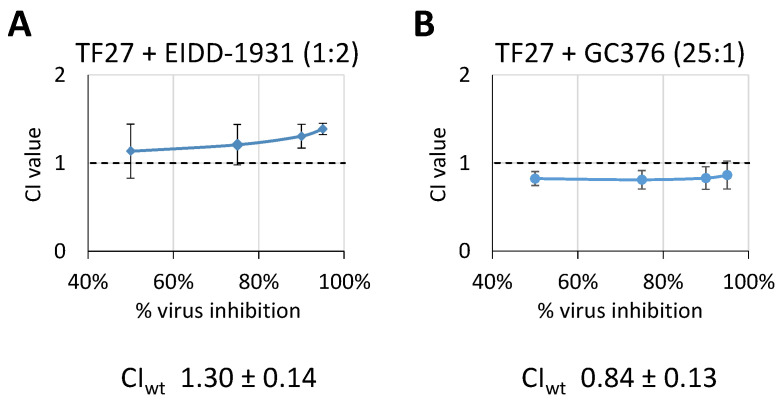
Combinatorial drug assessment of TF27 combinations with either EIDD-1931 or GC376 using the Loewe additivity fixed-dose method. Caco-2 cells were infected with SARS-CoV-2 d6-YFP at the MOI of 0.003 and treated with TF27, EIDD-1931, or GC376, either applied as single-drug treatment or as drug combinations of (**A**) TF27 + EIDD-1931 or (**B**) TF27 + GC376. Concentrations were matched for their respective EC_50_ values, starting at 4 × EC_50_ concentrations followed by seven twofold dilutions. The ratio of the compound concentrations is indicated in parenthesis. Viral replication was quantitated by measuring the cell-associated YFP fluorescence 28 h p.i. to determine dose responses. Subsequently, combination index (CI) values were calculated for 50, 75, 90, and 95% virus inhibition using the CompuSyn algorithm. The values represent the mean ± SD derived from three biological replicates.

Viral resistance formation represents a general problem when dealing with DAAs, which can be prevented by antiviral drug combination therapy. In the case of using two or more drugs, more pronounced antiviral activities are achievable. This reveals true synergistic antiviral activity which is defined by a combined effect that exceeds the sum of activities exerted by the corresponding single drugs. Interestingly, several reports have already described the promising synergistic drug interactions of ART with diverse anti-HCMV agents in vitro [52,53,54,55,56,57,58]. To investigate if TF27 exhibits synergism with approved as well as experimental SARS-CoV-2 antivirals, TF27′s drug interactions were analyzed by applying the Loewe additivity fixed-dose assay according to previously established protocols for SARS-CoV-2 infection [22]. As combination partners for TF27, EIDD-1931 (the active metabolite of molnupiravir), and GC376, (as a representative of 3CLpro-targeted protease inhibitors) were analyzed. These two drugs were selected based on their presumably distinct molecular action compared with ART-like compounds. The drug interactions were evaluated by the Loewe method which compares EC_50_, EC_75_, EC_90_, and EC_95_ values of single compounds with the corresponding EC values of a fixed-dose combination. From these results, combination indices (CI) for 50%, 75%, 90%, and 95% of inhibition were derived, termed CI_50_, CI_75_, CI_90_, and CI_95_, respectively [8]. CI values indicate additivity between 0.90 and 1.10, whereas synergy is assumed below and antagonism is assumed above this interval [43,59]. The overall drug interaction is described by the weighted CI value (CI_wt_) which favors the desirable near-complete inhibition of replication. Interestingly, the combination of TF27 with GC376 revealed a moderately synergistic drug interaction with constant CI values around 0.85 in the entire 50% to 95% inhibition interval and the resulting CI_wt_ of 0.84 ± 0.13 (Figure 5B). The combined treatment of TF27 and EIDD-1931 resulted in an antagonistic tendency, i.e., CI values >1, which became especially evident when approaching 95% of virus inhibition. This finding was also reflected by the CI_wt_ of 1.30 ± 0.14 indicating an overall moderately antagonistic relation (Figure 5A). Thus, the two selected examples demonstrated that drug combinations with TF27 exhibit unique characteristics of drug interaction, which in the case of 3CLpro inhibitors potentially provide an improved antiviral efficacy.

## 4. Discussion

### 4.1. The Broad-Spectrum Antiviral Potential of Monomeric ART and Trimeric TF27 and Their Putative Relevance for the Development of Novel Anti-SARS-CoV-2 Treatment Strategies

Researchers throughout the world have recently gained huge interest in the anti-SARS-CoV-2 potential of the approved and well-tolerated anti-malarial drugs from the artemisinin family, so that repurposing approaches for other infectious diseases appear generally appealing. Several in silico studies predicted ART to bind SARS-CoV-2 proteins, including the S protein, the viral proteases PLpro and 3CLpro by molecular docking and molecular dynamics simulations (reviewed in [60]). Early cell culture-based studies demonstrated ART anti-SARS-CoV-2 activities, however, at concentrations not achievable in humans or that are close to the in vitro cytotoxic concentrations [33,34,35,36,37]. These findings are consistent with our observations for the antiviral activity of monomeric ART. However, the trimeric TF27 revealed a very substantial antiviral activity indicating that trimerization greatly enhances the anti-SARS-CoV-2 activity. Additionally, TF27 potently inhibited SARS-CoV-2 replication in Calu-3 cells as well as delta and omicron replication in Caco-2 cells at low micromolar to submicromolar concentrations. Altogether, by using four different SARS-CoV-2 strains or variants in the context of two colon- and lung-derived human cell types, we demonstrated (i) the general high-level antiviral activity of TF27 when compared to ART and (ii) broad-spectrum anti-SARS-CoV-2 activity particularly for TF27. This relation of activity for ART and TF27 is consistent with earlier observations for HCMV and Marek’s disease virus (MDV), an oncogenic avian alphaherpesvirus, where the increase in TF27 over ART was 113-fold and >68 fold, respectively [10,19,20]. Regarding induction of cytotoxicity, although even 100 µM TF27 did not reduce cell viability below 50% according to the NRA in Caco-2 cells, a plateau at approx. 75% viability at concentrations between 0.4 µM and 12.5 µM was revealed. However, the LDH performed in the context of the MRA to specifically address cellular damage was inconspicuous at the same concentrations. These observations strongly indicate that TF27 exerts an antiproliferative effect on Caco-2 cells. The resulting selectivity indices for TF27 range between 29 and >204 dependent on the respective method/readout used. In comparison, using Calu-3 cells, neither ART nor TF27 revealed any significant reduction in cell viability up to 100 µM.

### 4.2. Mechanistic Properties Based on the Host-Directed Mode of Antiviral Activity of Trimeric TF27, Parental ART, and Related Compounds

From a mechanistic perspective, both ART and TF27 were similarly effective if the cells were only incubated with compounds prior to infection compared to the conventional setting, in which compounds were added concurrent with the viral inoculum. This finding further confirms cellular targets as primarily responsible for the inhibitory effect. Additionally, the highly reproducible but transitory increase in replication found uniquely for TF27-treated Calu-3 cells, but not observed in any other setting, is also consistent with an HDA profile. Combined treatment before and during infection substantially strengthened the antiviral EC_50_ for TF27 down to 0.06 µM and even revealed a clearly measurable degree of antiviral activity for ART. The duration in the presence of the compounds positively correlates with the antiviral effect. This might particularly explain the apparent increase in sensitivity of the omicron variant, since its delayed replication kinetics require longer incubation times in the respective replication assays. Translated into clinical settings, the prophylactic efficacy observed for the optimized ART derivative TF27 might, with intensified research and drug development, overcome the gap left by the currently approved DAAs that are not showing prophylactic properties so far.

### 4.3. The Chances of Nominating a New Candidate for Studying Antiviral Properties in Clinical Settings and the Question of TF27-Induced Viral Drug Resistance

The aim of this study has not been a nomination of TF27 for clinical candidate trials. Instead, the main intention of the investigations was to further explore the use of this model drug to gain closer insights into mechanistic details of its broad antiviral activity. Some limitations in solubility and the lack of a determination of eADME parameters have hampered swifter progression so far. It should be emphasized, however, that previous in vivo assessments of the anti-herpesviral efficacy of TF27 in the mouse and chicken models provided an important antiviral proof-of-concept [12,26,27]. These finding, together with the results of the present report, may provide a very valuable basis for the generation of even better suited derivatives of this class of trioxane/sesquiterpene drugs for future clinical development. Importantly, in addition to the benefits of prophylactic activity, the specific advantages of the targeting of host cell factors through host-directed antivirals (HDAs) may circumvent resistance formation. Although the possibility of viral resistance formation even against HDAs cannot be completely ruled out (possibly mediated through mutation of viral proteins directly interacting with the host targets of HDAs), the frequency in the appearance of such events is considered much lower than in the case of DAAs.

### 4.4. The Potential Benefit of TF27 as Part of a Combination Therapy to Achieve Improved Antiviral Efficacy by Exploiting Synergistic Drug Interactions

Besides prophylactic use, combination treatment represents a promising strategy to enhance drug efficacy. For SARS-CoV-2, HDA–DAA combinations as well as combinations of two HDAs with distinct targets often exert synergistic drug interactions [61,62,63,64,65,66,67]. Notable examples of well-understood anti-SARS-CoV-2 synergistic HDA–DAA pairs are the combinations DHODH inhibitors with nucleoside analogs [68,69,70,71]. In contrast, a study evaluating a more diverse set of 32 anti-SARS-CoV-2 compounds and combinations of HDAs and DAAs as well as of two DAAs revealed no clear pattern for their type of interactions, with some even switching the type depending on the investigated concentrations [72]. Consequently, even for mechanistically well-characterized drugs, models explaining their behavior in the context of drug combinations cannot always be easily derived. Considering all the different levels on which drug interactions potentially occur, it is difficult to explain the moderately antagonistic or synergistic interactions of TF27 with EIDD-1931 or GC376, respectively. Nevertheless, a drug with properties similar to TF27 as part of a combination treatment with 3CLpro inhibitors might address viral resistance formation and improve clinical outcomes.

Taken together, the pronounced anti-SARS-CoV-2 activity of TF27 adds to the notion of the outstanding properties of this investigational model drug and the broad antiviral activity of ART-like compounds. This may also open up further options of combination and prophylactic treatment schemes, possibly adding towards novel strategies of anti-SARS-CoV-2 therapy and prevention.

## Figures and Tables

**Table 1:** Broad-spectrum antiviral activity of ART and ART-derived compounds including the highly active trimeric derivative TF27.

Trioxane Compounds	Type of Investigation	Antiviral Activity Analyzed	References
Artesunate and artemisinin	Review article	Various anti-herpesviral and non-herpesviral activities	[6]
Artesunate	HHV-6A in cultured cells	Anti-HHV-6 activity	[15]
Artesunate	EBV reporter system	Anti-EBV activity	[11]
Artesunate and derivatives	Antiviral/mechanistic study	Broad-spectrum and NK-κB targeting	[10]
Hybrid compounds	Chemistry, confocal imaging	Anti-HCMV and intracellular trafficking	[39]
Trimers, dimers, and monomers	Antiviral/mechanistic study	TF27 unique, and strongest anti-HCMV drug	[19]
TF27, analogs/dendrimers	Comparing bioactivities	TF27 strongest antiviral activity, and target ID	[18]
TF27	MDV/chicken model	Inhibits MDV replication and tumorigenesis	[12]
TF27	MCMV/mouse model	Intraperitoneal MCMV treatment efficacy	[26]
TF27	cCMV ex vivo model	Anti-cCMV high efficacy	[40]
TF27	MCMV/mouse model	Anti-MCMV oral prophylactic efficacy	[27]
Autofluorescent BG95	Confocal imaging a.o.	Anti-HCMV and mitochondrial targeting	[41]
Linker model compounds	Target ID and verification	Mitochondrial/regulatory proteins as targets	[42]
TF27	Drug combination assessment	No true synergy TF27 + GCV	[43]
TF27 compared to artesunate	Multi-readout system	Strong anti-SARS-CoV-2 activity	Present study

**Table 2:** Comparative summary of in vitro antiviral activity, cytotoxicity, selectivity indices (SI), and fold increase in antiviral activity relative to the parental ART obtained for TF27 in HCMV and SARS-CoV-2 replication systems. The anti-SARS-CoV-2 EC_50_ for TF27 represents the mean values of eight independent experiments based on YFP quantitations. EC_50_ and CC_50_ values for ART and TF27 in the HCMV/HFFs in vitro model have been reported earlier [10,20]. n.d., not determined.

	HCMV/HFFs				SARS-CoV-2/Caco-2			
	EC_50_ [µM]	CC_50_ [µM]	SI	Fold Increase Relative to ART	EC_50_ [µM]	CC_50_ [µM]	SI	Fold Increase Relative to ART
**ART**	5.4 ± 0.6	>10	>2	1	>40	>100	n.d.	1
**TF27**	0.04 ± 0.01	>10	>250	113	0.46 ± 0.20	>100	>185	>87

## Data Availability

Not applicable.

## Supplementary Materials

The following supporting information can be downloaded at: https://www.mdpi.com/article/10.3390/pharmaceutics15010115/s1, Supplementary Methods.
