# Supplementary material for: The Trimeric Artesunate Analog TF27, a Broadly Acting Anti-Infective Model Drug, Exerts Pronounced Anti-SARS-CoV-2 Activity Spanning Variants and Host Cell Types
Source: Pharmaceutics. 2022 Dec 29;15(1):115. doi: 10.3390/pharmaceutics15010115 (PMC9866877; doi:10.3390/pharmaceutics15010115)
Supplement: Supplementary file 1 [file pharmaceutics-15-00115-s001.zip › pharmaceutics-2043088-supplementary.pdf]

Supplementary material

The Trimeric Artesunate Analog TF27, a Broadly Acting Anti-infective Model Drug, Exerts Pronounced Anti-SARS-CoV-2 Activity Spanning Variants and Host Cell Types

Friedrich Hahn [1,*], Christina Wangen [1], Sigrun Häge [1], Lars Herrmann [2], Alexandra Herrmann [1,3], Svetlana B. Tsogoeva [2] and Manfred Marschall [1,*]

[1] Institute for Clinical and Molecular Virology, Friedrich-Alexander University of Erlangen-Nürnberg (FAU), Schlossgarten 4, 91054 Erlangen, Germany

[2] Organic Chemistry Chair I and Interdisciplinary Center for Molecular Materials (ICMM), Friedrich-Alexander University of Erlangen-Nürnberg, Nikolaus-Fiebiger-Straße 10, 91058 Erlangen, Germany
[3] Immunic AG, Lochhamer Schlag 21, 82166 Gräfelfing, Germany
* Correspondence: friedrich.hahn@uk-erlangen.de (F.H.); manfred.marschall@fau.de (M.M.); Tel.: +49-9131-8536480 (F.H.); +49-9131-8526089 (M.M.)

Supplementary Methods

SARS-CoV-2 variants of concern, viral reporter recombinants, fluorescence-based and multi-readout replication assays

Four SARS-CoV-2 strains/recombinants were used in this study, i.e. the Wuhan-like wild-type Munich isolate (MUC-IMB-1/2020), variants of concern (VOCs) delta and omicron, and the B.1-derived recombinant reporter virus d6-YFP, in which the viral ORF6 was replaced by an enhanced yellow fluorescent protein (YFP)-encoding gene [22, 29]. The primary sequence of d6-YFP differs from the Wuhan wild-type isolate by five replacement mutations, i.e. 5'-UTR-T241C, nsp2-T265I, nsp12-P315L, S-D614G, and ORF3a-Q57H.

As the primary screening system, Caco-2 cells were inoculated with the SARS-CoV-2 recombinant d6-YFP. Infection settings were performed at the MOI of 0.003 in the presence of compounds. After 28 h, cells were fixed with 10% formalin and viral replication was determined by quantitation of the cell-associated YFP fluorescence in a Victor X4 microplate reader (PerkinElmer, Waltham, MA, USA). Infection of Calu-3 cells with d6-YFP was carried out with the MOI of 0.003, as determined on Caco-2 cells, with a 72-h period of replication. The DNA stain SYTOX Blue (ThermoFisher) was added as an internal control for the integrity of the fixed cell layer in Victor X4 measurements.

In specific cases, antiviral activity was determined by addressing several parameters of the SARS-CoV-2 replication by our previously established multi-readout assay (MRA, [22]. To this end, replication-associated antigens were detected in formalin-fixed permeabilized cells using subsequent antibody staining procedures. Particularly, the mouse monoclonal antibody mAb-S TRES-6.18 recognizing the viral spike (S) protein [22, 30] and the double-strand RNA-specific mAb J2 (SCIONS) [44] in combination with the secondary antibody anti-mouse Alexa 488 (A11029, ThermoFisher Scientific), were applied for the detection of viral gene products. For the respective quantitation of antibody-specific Alexa 488 signals, which had to be distinguished from the cellular YFP fluorescence originating from the infection with d6-YFP, fluorescent Alexa 488 antibody conjugates were eluted by incubation with 62.5 mM Tris pH 6.8, 100 mM β-mercaptoethanol and 2% SDS at 37 °C for 2 h [22].

Next, the eluted Alexa 488 conjugates were transferred to a separate multiwell plate, before measurement was carried out using the Victor X4 multilabel reader. After several washing steps with PBS, the cell layers were reused for additional rounds of antibody staining.

The optimized method for determination of viral replication by RT-qPCR directly from inactivated and proteinase K-treated cell culture supernatants has been described previously [22]. Briefly, viral supernatants were heat-inactivated, subsequently proteinase K-treated and 10fold diluted. The resulting samples were analyzed by RT-qPCR (NEB Luna Universal Probe One-Step RT-qPCR, E3006, NEB) following the manufacturer's instructions. Oligonucleotides were purchased from Biomers.net (Ulm, Germany) with sequences adapted from Corman et al., 2020 [31], using the primers RdRp_SARSr-F and RdRp_SARSr-R as well as the probe RdRp_SARSr-P2, however with Black Hole Quencher 1 (BHQ-1) as the 3′ quencher. Replication experiments with the clinical isolate MUC-IMB-1/2020 were carried out using the MOI of 0.003 followed by 28 h replication before fixation of the cells using formalin. The delta and omicron variants were obtained by cultivating 100 µL of anonymized residual patient swap samples on confluent Caco-2 cells. The identity of each variant was confirmed by mutation-specific RT-qPCR (Novaplex™ SARS-CoV-2 Variants VII Assay, Seegene, Düsseldorf, Germany) and Illumina-based next generation sequencing using MiSeq reagent kit v2 on a MiSeq™ instrument (Illumina, San Diego, CA, USA). Caco-2 cells were infected with an appropriate viral inoculum titrated in advance to result in approx. 75% infected cells at the time point of harvest. Replication was allowed to proceed for 48 h (delta) or 72 h (omicron) and terminated by formalin fixation. Viral infection rates of the unlabeled virus isolates were measured in permeabilized cells as described above by either staining with pAb-SARS-CoV-2-nsp3 (PA5116947, ThermoFisher Scientific) for the omicron and delta variants or mAb-S TRES-6.18 in the case of MUC-IMB-1/2020. The inhibitory activity of compounds on the SARS-CoV-2 replication in human cell lines was determined as described earlier [22, 29]. Antiviral activity was defined as the reduction of virus replication in compound-treated cells relative to solvent-treated control cells (DMSO), resulting in arithmetic mean values ± SD of biological quadruplicates. All SARS-CoV-2 infection experiments were performed under BSL-3 conditions.

Determination of cell viability by Neutral Red uptake assay and lactate dehydrogenase release assay

Compound-induced cytotoxicity in Caco-2 and Calu-3 cells was defined as a reduction of cell viability measured by Neutral Red uptake assay (NRA) according to previously established protocols [22, 32]. Briefly, media of cells treated with compounds for 48 h or 72 h, depending on the duration of the corresponding infection assay, were then adjusted to a final concentration of 40 µg/mL Neutral Red (Sigma Aldrich, St. Louis, MO, USA) followed by a 2 h incubation. Cell viability was determined based on the amount of incorporated Neutral Red after its release from the cells by addition of destaining solution (50% ethanol, 49% H_2O, 1% acetic acid) with subsequent fluorescence measurement at 560/630 nm for excitation/emission, respectively. The release of cellular lactate dehydrogenase (LDH) into the cell culture supernatant was measured using the CytoTox 96® Non-Radioactive Cytotoxicity Assay (Promega, Madison, WI, USA) according to the manufacturer's protocol. Values were normalized to maximal release of LDH using lysates of solvent-treated control cells as reference. Cell viability was defined as the absence of cytotoxicity.

[22] Hahn, F.; Hage, S.; Herrmann, A.; Wangen, C.; Kicuntod, J.; Jungnickl, D.; Tillmanns, J.; Muller, R.; Fraedrich, K.; Uberla, K.; Kohlhof, H.; Ensser, A.; Marschall, M. Methodological Development of a Multi-Readout Assay for the Assessment of Antiviral Drugs against SARS-CoV-2. *Pathogens* **2021**, *10*, doi:10.3390/pathogens10091076.

[29] Herrmann, A.; Jungnickl, D.; Cordsmeier, A.; Peter, A.S.; Uberla, K.; Ensser, A. Cloning of a Passage-Free SARS-CoV-2 Genome and Mutagenesis Using Red Recombination. *Int J Mol Sci* **2021**, *22*, doi:10.3390/ijms221910188.

[30] Peter, A.S.; Roth, E.; Schulz, S.R.; Fraedrich, K.; Steinmetz, T.; Damm, D.; Hauke, M.; Richel, E.; Mueller-Schmucker, S.; Habenicht, K.; Eberlein, V.; Issmail, L.; Uhlig, N.; Dolles, S.; Gruner, E.; Peterhoff, D.; Ciesek, S.; Hoffmann, M.; Pohlmann, S.; McKay, P.F.; Shattock, R.J.; Wolfel, R.; Socher, E.; Wagner, R.; Eichler, J.; Sticht, H.; Schuh, W.; Neipel, F.; Ensser, A.; Mielenz, D.; Tenbusch, M.; Winkler, T.H.; Grunwald, T.; Uberla, K.; Jack, H.M. A pair of noncompeting neutralizing human monoclonal antibodies protecting from disease in a SARS-CoV-2 infection model. *Eur J Immunol* **2022**, *52*, 770-783, doi:10.1002/eji.202149374.

[44] Schönborn, J.; Oberstrass, J.; Breyel, E.; Tittgen, J.; Schumacher, J.; Lukacs, N. Monoclonal antibodies to double-stranded RNA as probes of RNA structure in crude nucleic acid extracts. *Nucleic acids research* **1991**, *19*, 2993-3000, doi:10.1093/nar/19.11.2993.

[31] Corman, V.M.; Landt, O.; Kaiser, M.; Molenkamp, R.; Meijer, A.; Chu, D.K.; Bleicker, T.; Brunink, S.; Schneider, J.; Schmidt, M.L.; Mulders, D.G.; Haagmans, B.L.; van der Veer, B.; van den Brink, S.; Wijsman, L.; Goderski, G.; Romette, J.L.; Ellis, J.; Zambon, M.; Peiris, M.; Goossens, H.; Reusken, C.; Koopmans, M.P.; Drosten, C. Detection of 2019 novel coronavirus (2019-nCoV) by real-time RT-PCR. *Euro Surveill* **2020**, *25*, doi:10.2807/1560-7917.ES.2020.25.3.2000045.

[32] Repetto, G.; del Peso, A.; Zurita, J.L. Neutral red uptake assay for the estimation of cell viability/cytotoxicity. *Nat Protoc* **2008**, *3*, 1125-1131, doi:10.1038/nprot.2008.75.
